# Safranal, a Natural Neuroprotective Compound, Modulates Plasma Iron Levels, Improves Behavioral Performance, and Demonstrates Dose-Specific Cytoprotection in Alzheimer’s Disease Models

**DOI:** 10.3390/ijms27209392

**Published:** 2026-10-21

**Authors:** Esra Tekin, Meliha Koldemir Gündüz, Zeynep Yığman, Beyza Aydoğan Kara, Ayşegül Küçük, Mustafa Arslan

**Affiliations:** 1 Department of Physiology, Faculty of Medicine, Kırıkkale University, 71450 Kırıkkale, Türkiye; 2 Department of Engineering Basic Sciences, Faculty of Engineering and Natural Sciences, Kutahya Health Sciences University, 43100 Kutahya, Türkiye; meliha.koldemirgunduz@ksbu.edu.tr; 3 Department of Histology and Embryology, Faculty of Medicine, Gazi University, 06500 Ankara, Türkiye; zeynepyigman@gazi.edu.tr; 4 Neuroscience and Neurotechnology Center of Excellence (NÖROM), Gazi University, 06560 Ankara, Türkiye; 5 Department of Medical Services and Techniques, School of Health Services, Gazi University, 06830 Ankara, Türkiye; beyzaaydogan@gazi.edu.tr; 6 Life Sciences Application and Research Center, Gazi University, 06830 Ankara, Türkiye; mustafaarslan@gazi.edu.tr; 7 Department of Physiology, Faculty of Medicine, Bandırma Onyedi Eylül University, 10200 Bandırma, Türkiye; aysegul.kucuk@bandirma.edu.tr; 8 Department of Anesthesiology and Reanimation, Faculty of Medicine, Gazi University, 06500 Ankara, Türkiye; 9 Laboratory Animal Breeding and Experimental Research Center (GÜDAM), Gazi University, 06500 Ankara, Türkiye

**Keywords:** Alzheimer’s disease, safranal, metal homeostasis, intracerebroventricular streptozotocin, Sprague–Dawley rat, SH-SY5Y cell line

## Abstract

Metal dyshomeostasis contributes to Alzheimer’s disease (AD) by influencing amyloid aggregation, oxidative imbalance, and impaired clearance mechanisms. Safranal, a bioactive constituent of *Crocus sativus*, exhibits antioxidant properties, but its metal-modulating actions remain insufficiently understood. This study investigated whether safranal modulates metal (Fe, Cu, Zn) levels in an AD rat model while evaluating its cytoprotective rescue capacity against Aβ1-42 induced neurotoxicity in differentiated SH-SY5Y cells. AD was induced in male rats via intracerebroventricular streptozotocin, followed by a four-week safranal administration (0.5 mg/kg/day, ip). Behavioral performance, plasma metal levels (ICP-MS), and cerebral total antioxidant capacity (FRAP) were evaluated alongside histological assessment. Post-treatment protocols were employed to evaluate the effects of safranal on cell viability and apoptosis in differentiated SH-SY5Y cells exposed to Aβ1-42. Longitudinal analyses revealed that safranal attenuated exploratory deficits and recognition memory impairments in AD rats. Decreased plasma iron levels in the AD group were restored by safranal, accompanied by a return of cerebral antioxidant capacity to normal. Although overt hippocampal histological alterations were absent in this early phase, post-treatment with safranal successfully offered a therapeutic rescue window, preserving cell viability and attenuating Aβ1-42-induced apoptotic features in vitro. Safranal demonstrates beneficial neuroprotective effects in AD models by improving behavioral outcomes, partially correcting plasma iron levels, and mediating cytoprotective rescue windows against Aβ1-42-induced cellular toxicity. These findings suggest that safranal represents a promising natural candidate for reducing behavioral deficits, supporting peripheral iron status, and offering functional cell survival in AD models.

## 1. Introduction

Alzheimer’s disease (AD) is the most common cause of dementia and a major global health challenge, affecting millions of individuals worldwide. Despite extensive research efforts, currently available therapies provide only symptomatic relief and fail to prevent or reverse the underlying neurodegenerative processes [1,2]. The pathogenesis of AD is multifactorial and involves amyloid-β (Aβ) accumulation, tau pathology, oxidative stress, mitochondrial dysfunction, neuroinflammation, and progressive neuronal loss [3,4]. These interconnected mechanisms ultimately contribute to cognitive decline and impaired neuronal function [5].

Increasing evidence indicates that metal dysregulation plays an important role in AD pathogenesis [6]. Essential transition metals, particularly iron, copper, and zinc, are involved in numerous neuronal processes, including neurotransmitter synthesis, mitochondrial function, and antioxidant defense [7,8]. Disturbances in metal homeostasis have been associated with enhanced oxidative stress, abnormal protein aggregation, impaired neuronal metabolism, and increased vulnerability to neurodegeneration [9]. Among these metals, iron has attracted particular attention because its dysregulation promotes reactive oxygen species generation and may exacerbate neuronal injury through oxidative mechanisms [10,11]. Peripheral iron alterations are known to reflect systemic metabolic and redox changes that can influence central nervous system function, and several studies have reported parallel shifts between plasma and brain metal status in neurodegenerative conditions [12,13]. Therefore, assessment of plasma metals may provide a practical and biologically relevant indicator of metal-related dysregulation in AD models. Although metal accumulation in the brain is a feature of advanced-stage AD, it is thought that intracerebroventricular–streptozotocin (icv-STZ) administration can lead to early systemic metabolic changes and peripheral iron dysregulation prior to significant cerebral metal accumulation. The present study specifically focused on investigating whether safranal modulates peripheral trace metal levels (iron, copper, zinc) combined with cerebral total antioxidant capacity in the early phases of AD-like model.

Oxidative stress is considered a central contributor to AD progression. Excessive production of reactive oxygen species disrupts cellular homeostasis, damages biological macromolecules, and impairs neuronal survival [14]. Hence, compounds capable of reducing oxidative injury while supporting physiological metal balance have emerged as attractive therapeutic candidates for neurodegenerative disorders.

Safranal, a major volatile constituent of *Crocus sativus*, has been reported to possess antioxidant, anti-inflammatory, anti-apoptotic, and neuroprotective properties [15,16,17]. Experimental studies have demonstrated that safranal can improve cognitive performance, reduce oxidative damage, and protect neuronal cells from various neurotoxic insults [18,19]. Although the neuroprotective actions of safranal have been increasingly investigated, its potential role in alleviating metal dysregulation associated with AD remains largely unexplored.

To investigate these mechanisms, complementary in vivo and in vitro AD models were employed. The icv-STZ rat model reproduces several features of sporadic AD, including cognitive dysfunction, oxidative stress, and impaired cerebral metabolism [20,21]. In addition, Aβ1-42-treated SH-SY5Y cells provide a well-established experimental platform for examining the neuroprotective rescue capacities of potential agents [22,23].

**Figure 1:**
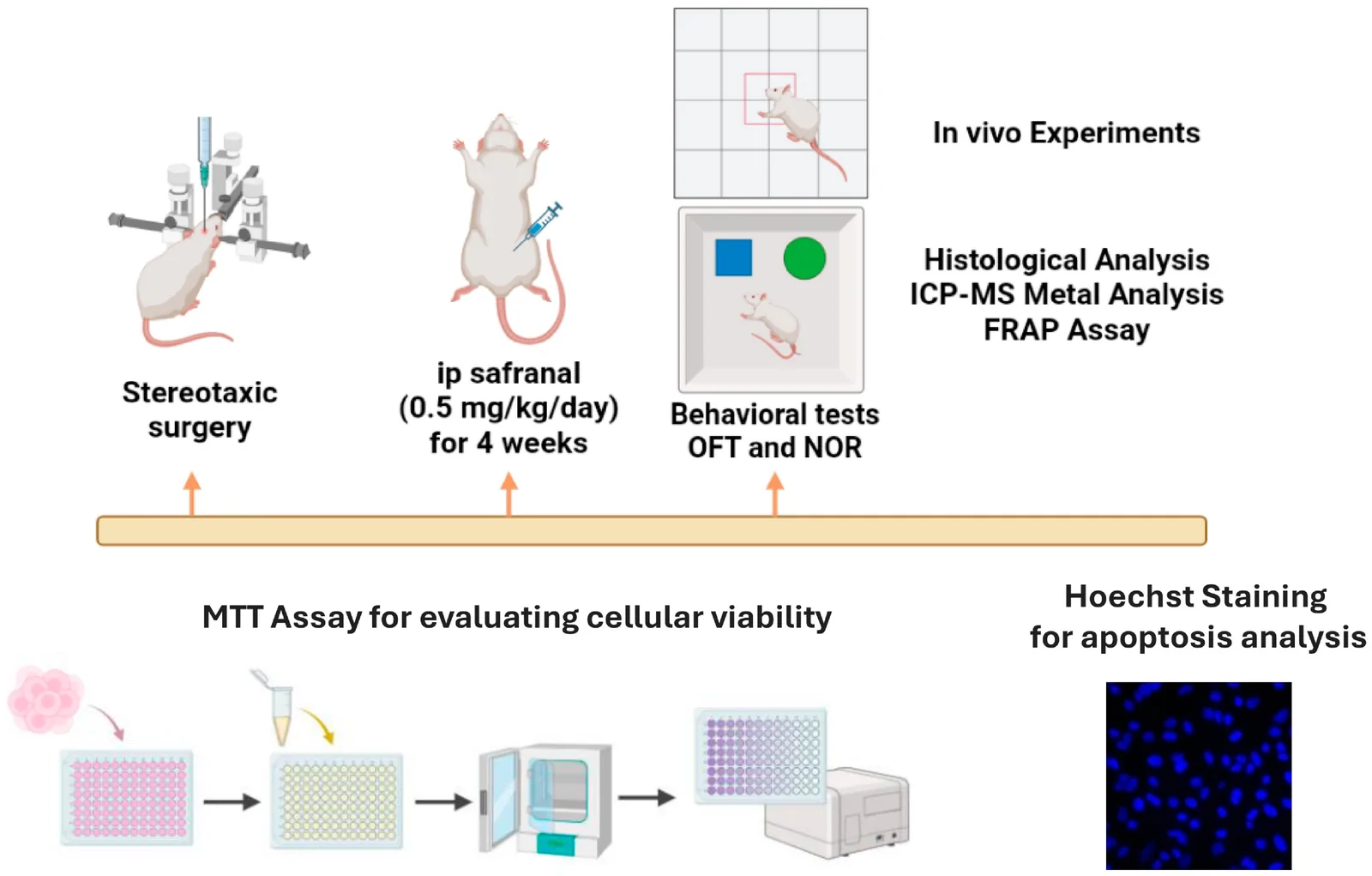
Schematic overview of the experimental workflow.

This study aimed to evaluate the potential cytoprotective rescue effects of safranal against Aβ-induced neurotoxicity, while investigating its potential to improve behavioral outcomes and cerebral total antioxidant capacity, as well as regulate plasma metal levels (iron, copper, zinc) in AD models. While the baseline neuroprotective capacity of safranal is well-documented [16,18,24], the present study specifically aimed to bridge a critical literature gap by investigating its novel modulatory actions on peripheral trace metal status, particularly plasma iron homeostasis, alongside cerebral antioxidant capacity in the early phases of AD-like model. Rather than modeling advanced neurodegeneration and irreversible tissue loss, our study targeted to reflect the early, metabolic stages of AD. The experimental design of this study, including stereotaxic surgery, safranal administration, behavioral tests, and in vivo and in vitro analyses, is summarized in Figure 1.

In the in vivo phase, rats underwent stereotaxic surgery followed by daily intraperitoneal (ip) safranal administration (0.5 mg/kg) for four weeks, after which behavioral assessments (Open Field Test [OFT] and Novel Object Recognition Test [NOR]) and samples were processed for histological evaluation, ICP-MS metal quantification, and FRAP antioxidant capacity analysis (in vivo phase: *n* = 7 rats per group). In the sequential in vitro phase, human SH-SY5Y neuroblastoma cells were initially subjected to a differentiation protocol using all-trans retinoic acid to develop complex neuritic networks. The cells were pre-exposed to a neurotoxic Aβ1-42 baseline for 48 h, followed by a 48 h incubation with graded concentrations of safranal to evaluate its dose-specific cytoprotective rescue windows. The apoptotic alterations were qualitatively validated via Hoechst 33258 nuclear fluorescent staining.

## 2. Results

### 2.1. Longitudinal Evaluation of Spontaneous Locomotor Activity and Anxiety-like Behavior in the Open Field Test (OFT)

To evaluate the neuroprotective potential of safranal against AD-induced behavioral deterioration, horizontal and vertical exploratory drive, grooming behaviors, and emotional reactivity were longitudinally assessed in the Open Field Test (OFT) at baseline and final time points.

For peripheral line crossings, the Two-Way Repeated Measures ANOVA demonstrated a significant Time × Group interaction [F(4, 30) = 3.093, *p* = 0.0303], demonstrating that the developmental trajectory of horizontal locomotion over time was strictly group-dependent. Subsequent Sidak’s post hoc test revealed that baseline peripheral activity was uniform across all experimental groups (*p* > 0.05, Table 1), while severe locomotor suppression emerged in the untreated Alzheimer’s group at the final endpoint (*p* < 0.05, Table 1), an impairment that was mitigated by safranal (*p* < 0.05, Table 1).

A parallel pattern was confirmed for central line crossings, which also demonstrated a significant Time × Group interaction [F(4, 30) = 3.175, *p* = 0.0274]. Additionally, significant main effects of Time [F(1, 30) = 8.194, *p* = 0.0076] and Group [F(4, 30) = 9.609, *p* < 0.0001] were captured. While baseline exploration of the central zone did not differ significantly among the groups (*p* > 0.05, Table 1), terminal assessments showed reduced central-zone exploration in the untreated Alzheimer’s group, characterized by significantly lower central crossing frequencies compared with the healthy control, sham, and safranal-alone groups (*p* < 0.05, Table 1). In contrast, the combined Alzheimer’s + safranal group exhibited an attenuation of these central exploratory deficits (*p* < 0.05, Table 1).

Vertical exploratory behaviors are presented in Table 2. For rearing frequency, a Time × Group interaction was detected [F(4, 30) = 4.443, *p* = 0.0061], accompanied by significant main effects of Time [F(1, 30) = 6.111, *p* = 0.0193] and Group [F(4, 30) = 4.695, *p* = 0.0046]. Similarly, rearing duration demonstrated a significant Time × Group interaction [F(4, 30) = 3.434, *p* = 0.0200] along with strong main effects of Time [F(1, 30) = 7.772, *p* = 0.0091] and Group [F(4, 30) = 4.595, *p* = 0.0052]. Post hoc analysis verified that while all groups displayed identical vertical exploration rates at baseline (*p* > 0.05, Table 2), the final trial revealed vertical exploration deficits in untreated Alzheimer’s rats compared to control (*p* < 0.05, Table 2). Safranal administration in the Alzheimer’s group resulted in a subtle, descriptive elevation in both vertical parameters, though this recovery trend did not reach statistical significance at the terminal endpoint (*p* > 0.05, Table 2).

For defecation scores, Sidak’s multiple comparisons test revealed that all experimental groups were statistically similar at baseline, with no significant differences observed between any of the groups (*p* > 0.05). The two-way RM ANOVA demonstrated that the Time × Group interaction did not reach statistical significance [F(4, 30) = 1.691, *p* = 0.1782], indicating that the patterns of change over time were statistically similar across all experimental groups. However, a significant main effect of Time was observed [F(1, 30) = 8.813, *p* = 0.0058], revealing a general decrease in defecation scores from baseline to the final assessment. Additionally, the main effect of Group did not reach statistical significance [F(4, 30) = 2.350, *p* = 0.0768], suggesting that overall defecation scores did not vary significantly among the groups throughout the study (Table 3).

For grooming frequency, the Time × Group interaction was non-significant [F(4, 30) = 1.267, *p* = 0.3047], with no significant main effects for Time or Group (*p* > 0.05, Table 4). Similarly, grooming duration showed an insignificant interaction [F(4, 30) = 0.6300, *p* = 0.6449] and an insignificant main effect of Group (*p* = 0.8596), although a significant main effect of Time was present [F(1, 30) = 11.13, *p* = 0.0023, Table 4]. Furthermore, parameters evaluating baseline versus terminal states of akinesia, including immobility duration [Interaction: F(4, 30) = 1.189, *p* = 0.3356, Table 3] and immobility frequency [Interaction: F(4, 30) = 0.7234, *p* = 0.5828, Table 3], yielded no statistically significant interactions or main effects (*p* > 0.05).

### 2.2. Recognition Memory Performance in the Novel Object Recognition (NOR) Test

To comprehensively evaluate cognitive flexibility, visual attention, and long-term recognition memory, the Discrimination Index (DI %) and Recognition Index (RI %) were systematically assessed across all experimental groups (Table 5).

For the Discrimination Index (DI %), a Two-Way Repeated Measures ANOVA (Group × Phase) was performed to evaluate differences in discrimination performance between the Identical Objects Phase and the Different Objects Phase across experimental groups. The analysis revealed a highly significant Phase × Group interaction effect [F(4, 30) = 8.132, *p* = 0.0001], indicating that changes in object discrimination performance across phases differed significantly among the groups. Significant main effects were also observed for both Phase [F(1, 30) = 46.10, *p* < 0.0001] and Group [F(4, 30) = 11.43, *p* < 0.0001]. Subsequent Sidak’s multiplicity-adjusted post hoc analysis demonstrated that DI scores during the Identical Objects Phase did not differ significantly among the experimental groups (*p* > 0.05), indicating comparable performance during the initial phase. Consistent with this, the Two-Way Repeated Measures ANOVA also showed no group differences in the identical objects phase (group effect *p* > 0.05), while the significant group × phase interaction and robust phase effect indicated that marked alterations emerged specifically during the Different Objects Phase.

The prominent group divergence emerged during the choice trial (Different Objects Phase). The untreated Alzheimer’s group exhibited a profound, statistically significant reduction in DI scores compared to the control, sham, and safranal-treated groups (*p* < 0.05). Safranal treatment in the Alzheimer’s + safranal group rescued this cognitive decay, inducing a statistically significant restoration of discrimination performance compared to the untreated Alzheimer’s group (*p* < 0.05), although this recovery remained below the control and sham baseline thresholds (*p* < 0.05).

For the Recognition Index (RI %), because this specific parameter is inherently localized to the choice trial containing the novelty stimulus (Different Objects Phase), data were analyzed via One-Way ANOVA followed by Tukey’s multiplicity-adjusted post hoc test. The One-Way ANOVA performed for the Recognition Index (RI %) revealed a significant group effect [F(4, 30) = 6.020, *p* = 0.001; MSE = 76.04]. Post hoc comparisons verified that untreated Alzheimer’s rats suffered from substantial memory deficits, exhibiting significantly lower RI values compared to the control, sham, and safranal-alone groups (*p* < 0.05). While safranal administration in the Alzheimer’s + safranal group demonstrated an upward trend and partially attenuated this cognitive decay, the enhancement did not reach full statistical significance against the untreated Alzheimer’s group (*p* > 0.05), remaining significantly below the control group (*p* < 0.05), as detailed in Table 5.

### 2.3. Histological Evaluation

**Figure 2:**
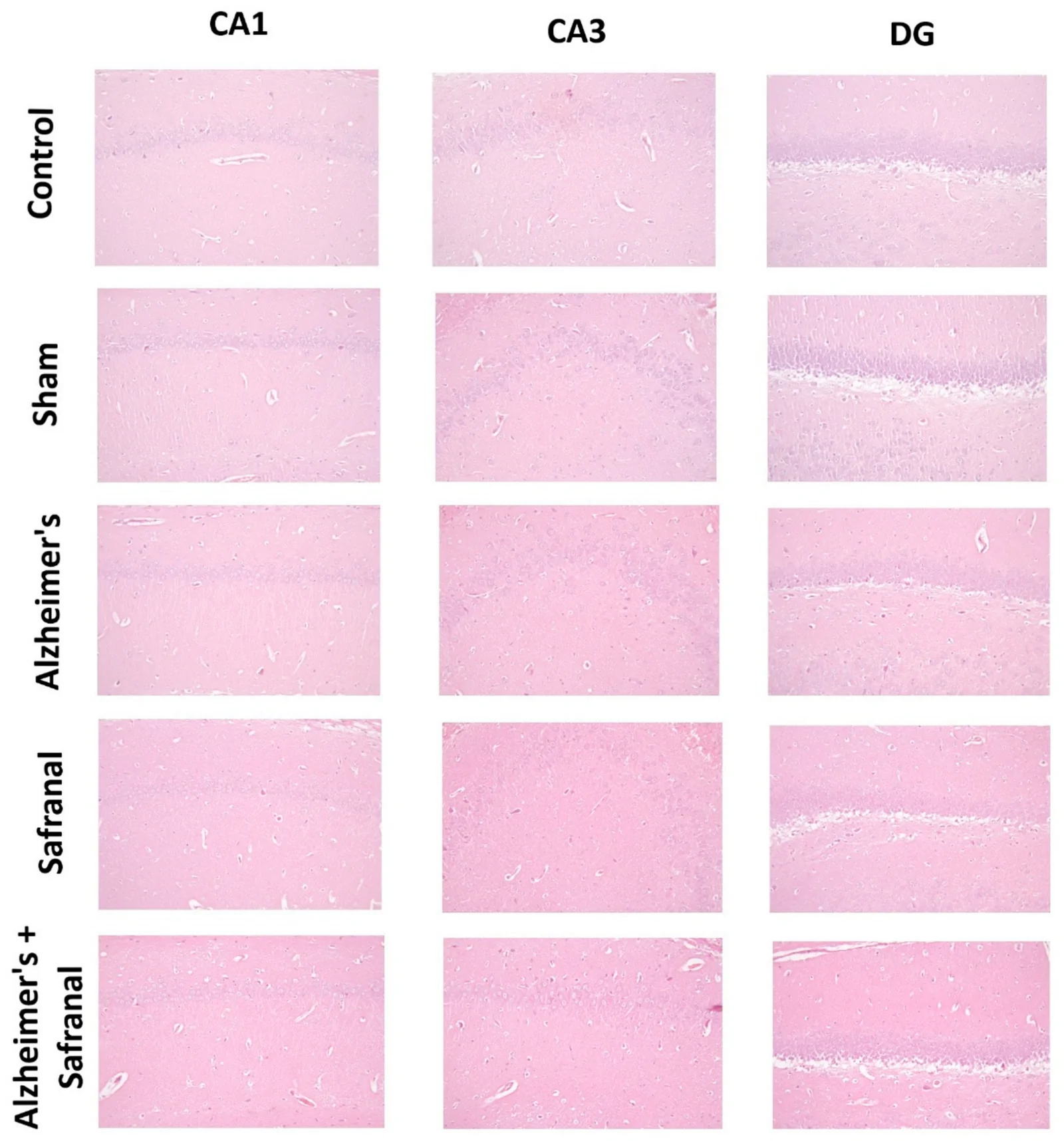
Hippocampal cornu ammonis 1 (CA1), cornu ammonis 3 (CA3), and dentate gyrus (DG) regions of the groups stained with Hematoxylin-Eosin (*n* = 7 per group).

Hematoxylin–eosin staining revealed preserved hippocampal cytoarchitecture across all experimental groups. Qualitative visual assessment demonstrated that neuronal morphology, laminar organization, and cellular density within the cornu ammonis 1 (CA1), CA3, and dentate gyrus (DG) regions remained structurally comparable, with no overt degenerative changes or macroscopic tissue loss observed (Figure 2).

**Figure 3:**
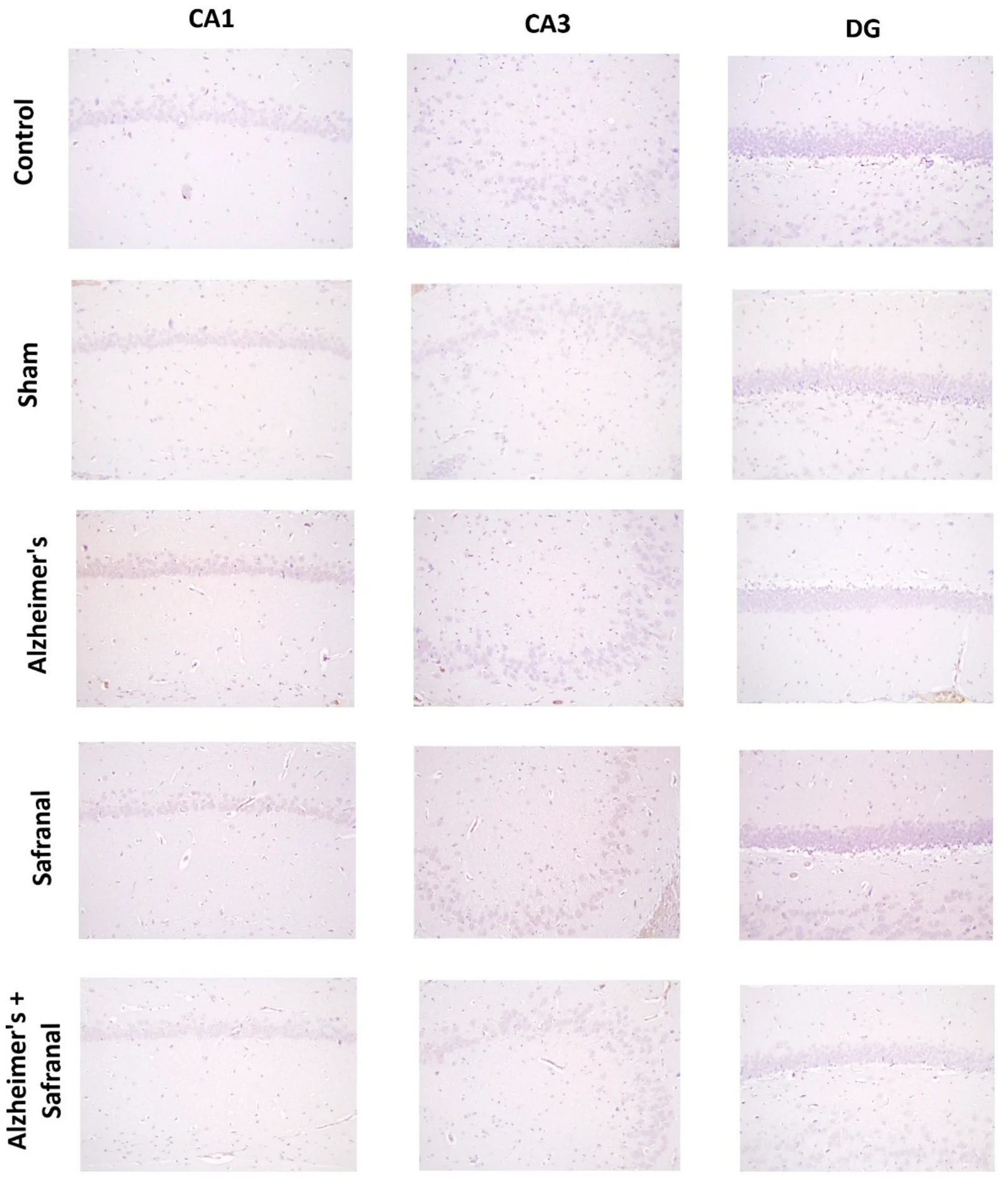
Representative immunohistochemical images obtained from the hippocampal cornu ammonis 1 (CA1), cornu ammonis 3 (CA3), and dentate gyrus (DG) regions using the MOAB-2 antibody (*n* = 7 per group).

Immunohistochemical staining with MOAB 2 was utilized for a qualitative visual assessment of Aβ immunoreactivity across the experimental groups. Microscopic inspection revealed a baseline level of diffuse background staining, with no detectable differences in staining intensity, pattern, or localized distribution within the CA1, CA3, and DG regions across any of the groups, and no overt extracellular plaque consolidation was observed (Figure 3).

### 2.4. Plasma Metal Levels (Inductively Coupled Plasma Mass Spectrometry [ICP-MS])

**Figure 4:**
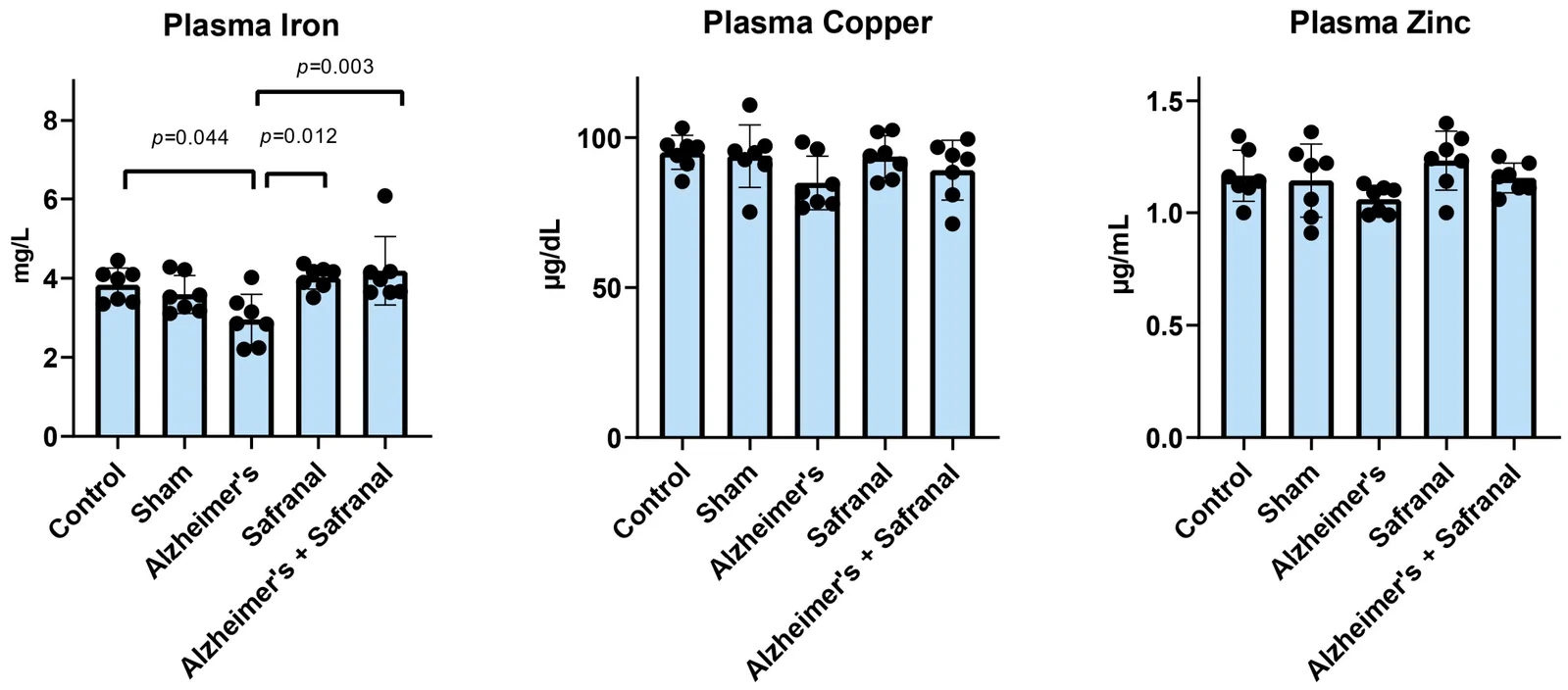
Iron, copper, and zinc concentrations obtained from rat plasmas using the ICP-MS method. Data are expressed as mean ± SD (*n* = 7 per group). Horizontal significance bars indicate statistical comparisons between groups evaluated via One-Way ANOVA followed by Tukey’s post hoc test, displaying multiplicity-adjusted exact *p*-values directly above the lines.

Plasma iron concentrations differed significantly among groups (*p* < 0.05). The Alzheimer’s group exhibited significantly reduced iron levels compared with control, safranal, and Alzheimer’s + safranal groups (*p* < 0.05). Safranal treatment restored iron concentrations to near-control levels (Figure 4). Copper and zinc concentrations showed a nonsignificant trend toward reduction in the Alzheimer’s group (*p* > 0.05).

### 2.5. Total Antioxidant Capacity (Ferric Reducing Antioxidant Power [FRAP Analysis])

**Figure 5:**
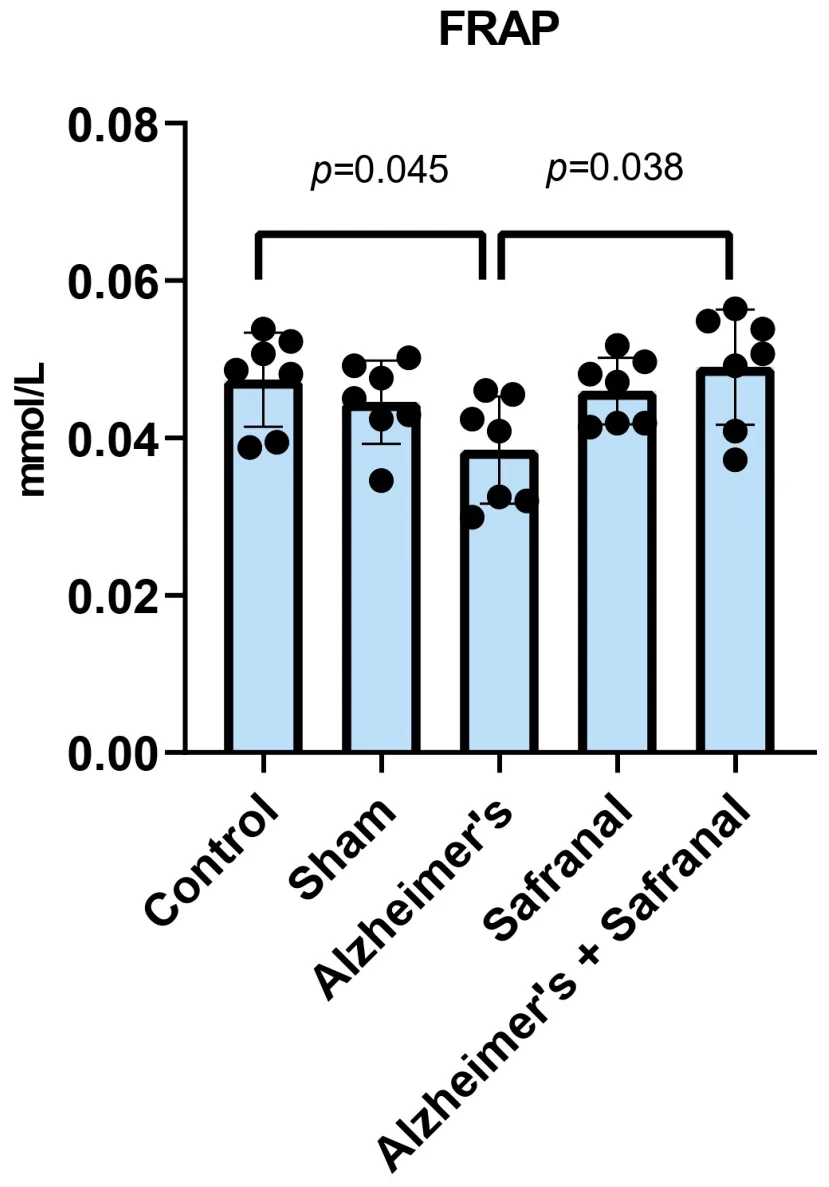
Brain total antioxidant capacity levels determined using the FRAP method. Data are expressed as mean ± SD (*n* = 7 per group). Horizontal significance bars indicate statistical comparisons between groups evaluated via One-Way ANOVA followed by Tukey’s post hoc test, displaying multiplicity-adjusted exact *p*-values directly above the lines.

FRAP analysis of brain tissue revealed significantly reduced antioxidant capacity in the Alzheimer’s group compared with control and Alzheimer’s + safranal groups (*p* < 0.05; Figure 5). Safranal treatment restored FRAP values toward normal levels.

### 2.6. In Vitro Cytoprotective Efficacy and Therapeutic Window of Safranal Against Aβ1-42-Induced Toxicity

**Figure 6:**
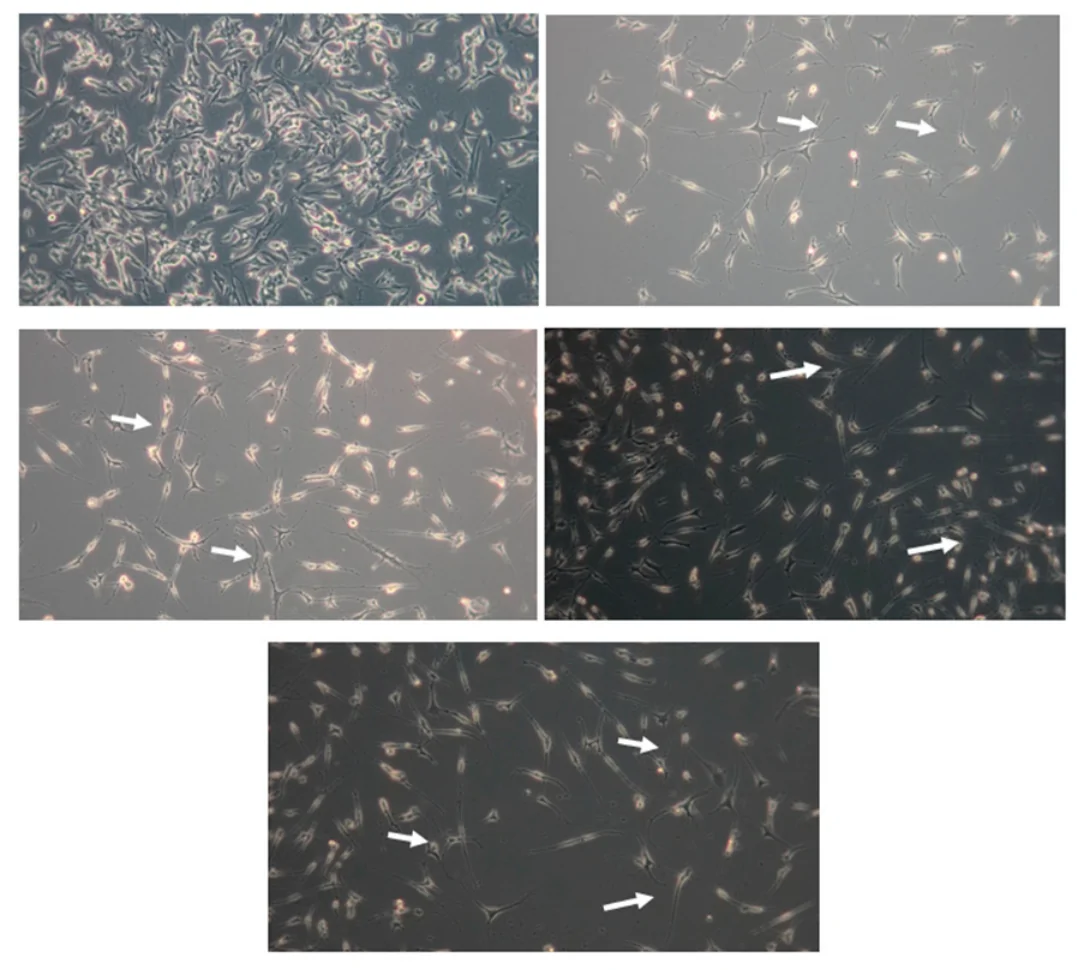
Morphology and network formation of SH-SY5Y neuroblastoma cells. Retinoic acid treatment induced the development of thin, branched neurites and supported network formation (arrows indicate neurites; 10× magnification).

**Figure 7:**
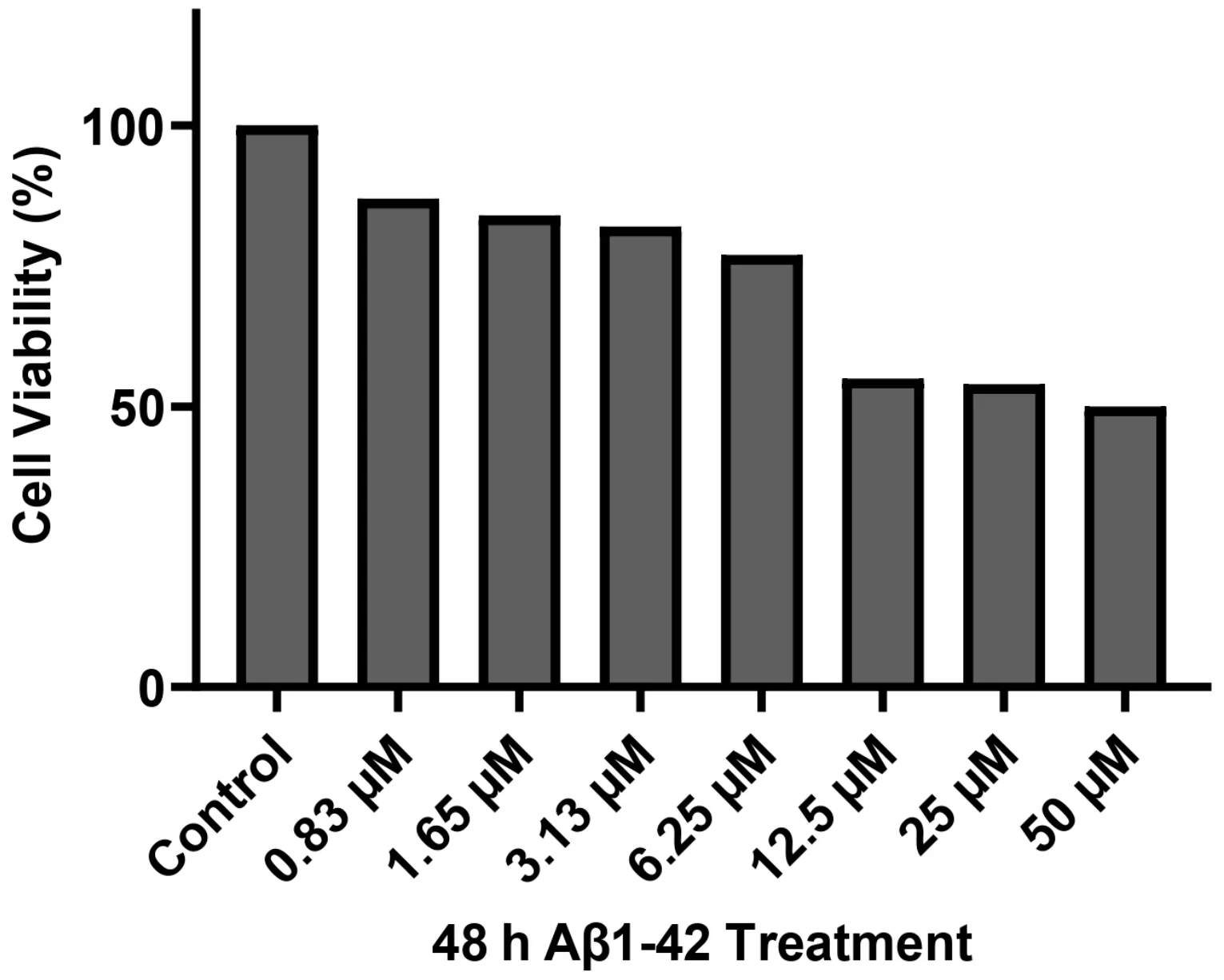
Preliminary concentration-dependent neurotoxic screening and cell viability profile of differentiated SH-SY5Y neuroblastoma cells exposed to graded concentrations of Amyloid-beta (Aβ1-42) for 48 h, quantified via the MTT assay. The screening outlines a concentration-dependent reduction trend in cellular survival compared to the untreated control group (100%), establishing the experimental baseline threshold for neurotoxic model verification. Data represent the baseline mean viability values normalized across triplicate screening wells.

**Figure 8:**
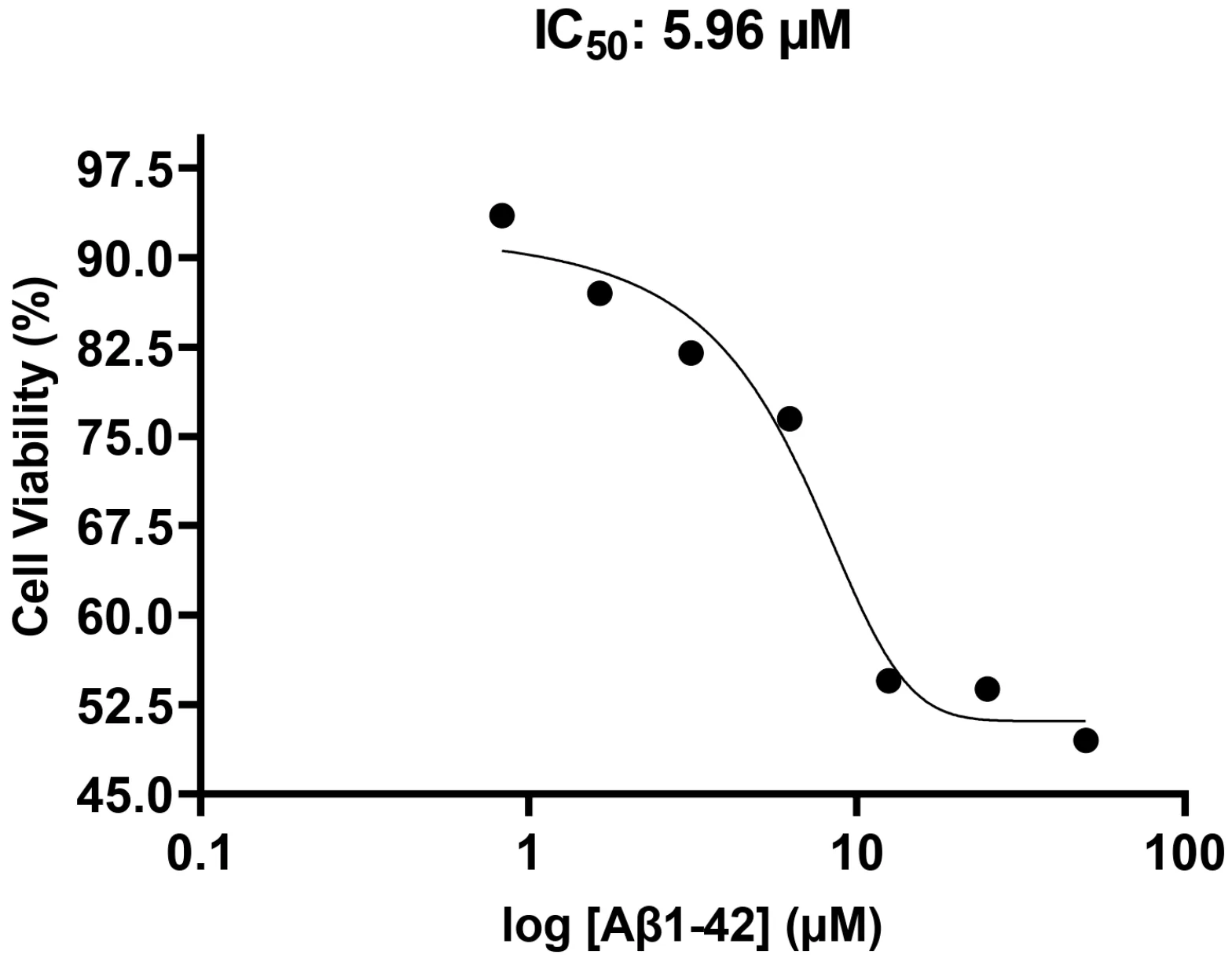
Non-linear regression curve and sigmoidal dose–response tracking of Amyloid-beta (Aβ1-42) induced neurotoxicity in differentiated SH-SY5Y neuroblastoma cells after 48 h of exposure. The calculated relative neurotoxic IC_50_ value was determined as 5.96 µM, serving as the experimental baseline validation for the establishment of the in vitro AD model. Data points represent independent computerized fitting models normalized against the untreated control group (100%).

Following successful retinoic acid differentiation, which promoted the extension of thin, branched neurites and complex cellular network formation (Figure 6), differentiated SH-SY5Y neuroblastoma cells were exposed to various concentrations of Aβ1-42 (0.83–50 µM) for 48 h to determine the neurotoxic threshold for model establishment. Exposure to Aβ1-42 induced a concentration-dependent reduction in cell viability (Figure 7), yielding a calculated relative neurotoxic IC_50_ value of 5.96 µM. The standalone non-linear regression curve tracking this neurotoxic baseline screening is detailed in Figure 8.

**Figure 9:**
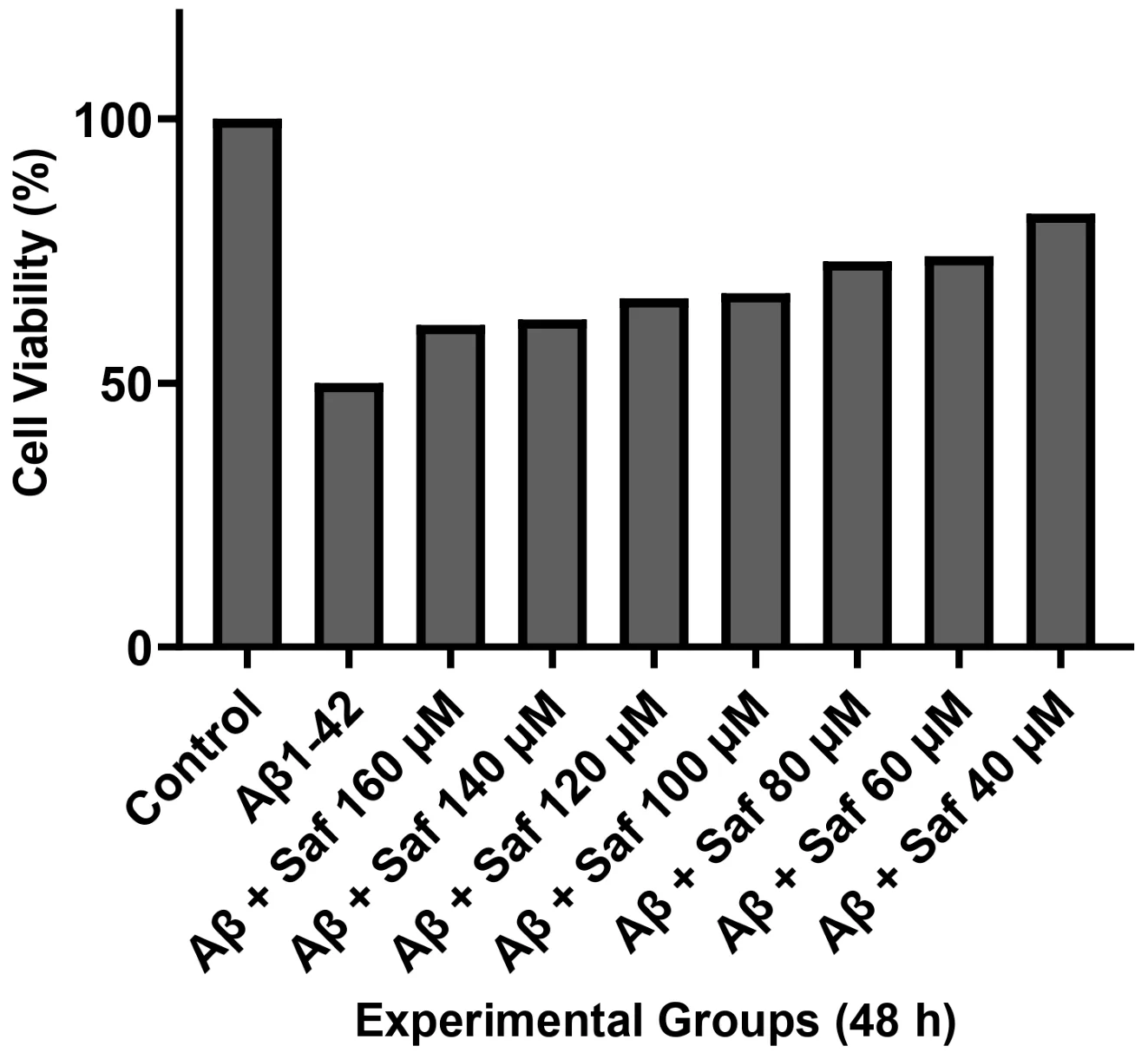
Dose-specific cytoprotective profile and cell viability tracking of Aβ1-42-pretreated differentiated SH-SY5Y neuroblastoma cells following 48 h of incubation with graded concentrations of safranal, quantified via the MTT assay. The screening outlines a therapeutic trend where lower concentrations of safranal (40–60 µM) rescue cells from Aβ1-42-induced toxicity, elevating survival from the ~50% pathological baseline up to 82%. Conversely, the decline in survival captured at higher concentration ranges of safranal demonstrates that exceeding the optimal therapeutic rescue window limits its beneficial efficacy within this post-treatment paradigm. Data represent the mean viability values normalized across triplicate screening wells (Saf, Safranal).

To evaluate the potential cytoprotective capacity and map the therapeutic rescue windows of safranal, Aβ1-42-pretreated differentiated SH-SY5Y cells were subsequently incubated with graded concentrations of safranal (40–160 µM) for an additional 48 h. The baseline screening outlined a dose-specific protective profile (Figure 9). At the lower concentration range (40–60 µM), safranal demonstrated a protective capacity, rescuing cell viability up to 82% (at 40 µM) and 74% (at 60 µM) compared to the unprotected Aβ1-42-only vehicle control baseline (~50%). Conversely, as safranal concentrations escalated into the higher range (120–160 µM), this cytoprotective rescue trend progressively declined, with cell survival tracking toward 66% and 61%, demonstrating that exceeding the optimal therapeutic rescue window limits its beneficial efficacy within this post-treatment paradigm.

### 2.7. Hoechst Apoptosis Analysis

**Figure 10:**
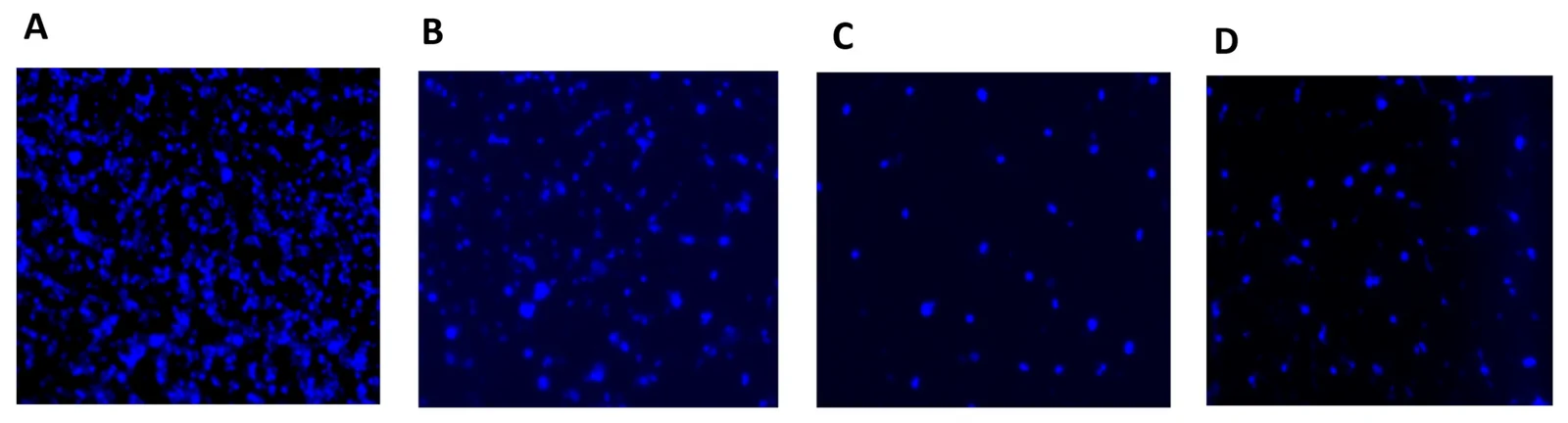
Qualitative evaluation of Hoechst 33258 nuclear fluorescent staining. The micrographs evaluate the cytoprotective effects of safranal against Aβ1-42-induced neurotoxicity and apoptotic changes in differentiated SH-SY5Y cells. Qualitative visual assessment of representative fluorescence microscopy images displays nuclear morphology across experimental conditions. (**A**) Undifferentiated control group showing dense, uniformly stained nuclei with rapid proliferation features. (**B**) Differentiated neuron-like control cells displaying typical structured, post-mitotic nuclear morphology. (**C**) Induced AD model group exposed to the Aβ1-42 neurotoxic baseline, characterized by severe cellular depletion, distinct nuclear condensation (intensely hyper-fluorescent shrunken spots), and apoptotic fragmentation. (**D**) AD model cells treated with lower concentrations of safranal, demonstrating marked morphological preservation of nuclear architecture, increased overall cell density, and resistance against amyloid-induced neurodegeneration.

As a qualitative morphological assessment of nuclear architecture, Hoechst 33258 staining revealed dense, intact nuclei in control cells and well-organized nuclear morphology in healthy differentiated neuron-like cells. Exposure to the designated Aβ1-42 neurotoxic baseline induced severe cell depletion alongside classical apoptotic hallmarks, including marked nuclear condensation and fragmentation characterized by intensely hyper-fluorescent, shrunken bright blue spots, thereby confirming robust apoptotic cellular decay. In strict alignment with the viability metrics, post-treatment with sub-toxic concentrations of safranal partially restored nuclear structural integrity, markedly attenuated chromatic fragmentation, and increased overall cell density compared with the unprotected Alzheimer’s vehicle group, displaying a reduction in condensed apoptotic remnants (Figure 10A–D).

## 3. Discussion

AD is a multifactorial neurodegenerative disorder in which metal dyshomeostasis, oxidative imbalance, mitochondrial dysfunction, and impaired clearance mechanisms interact to accelerate neuronal damage [3,4]. In this study, we investigated the neuroprotective potential of safranal, a natural constituent of *Crocus sativus*, using complementary in vivo and in vitro AD models. While our outcomes confirm the established neuroprotective and cytoprotective properties of safranal, the prominent finding of this study is the novel demonstration that safranal administration can modulate and partially restore altered plasma iron levels alongside cerebral FRAP capacity in an early-stage AD model.

The behavioral impairments observed in the icv-STZ model are consistent with previous reports describing reduced locomotor activity, exploratory behavior, and center zone usage in STZ-induced neurodegeneration [25,26]. STZ disrupts neuronal function through impaired glucose metabolism, cholinergic dysfunction, oxidative stress, and synaptic loss [27], which likely contributed to the decreased line crossings and rearing behavior in the Alzheimer’s group. Safranal treatment improved these behavioral deficits, restoring peripheral line crossings and triggering a descriptive but non-significant trend toward rearing recovery. These improvements may be associated with the antioxidant and neuroprotective properties of safranal, which have been reported in previous experimental studies [28,29]. No significant differences were observed in grooming, defecation, or immobility parameters, suggesting that these behavioral domains were less affected under the present experimental conditions.

The novel object recognition test further confirmed the cognitive impairment induced by STZ and the ameliorative effects of safranal. Reduced discrimination and recognition indices in the Alzheimer’s group are consistent with hippocampal dysfunction characteristic of STZ-induced AD [25]. Safranal partially restored recognition memory, indicating a beneficial effect on cognitive performance in the STZ-induced AD model. These findings are consistent with previous studies reporting neuroprotective and cognition-enhancing effects of safranal and other antioxidant interventions in experimental AD models [30,31].

Metal dysregulation is increasingly recognized as an important contributor to AD pathogenesis [32]. Essential metals such as iron, copper, and zinc participate in numerous physiological processes, including mitochondrial energy metabolism, neurotransmitter synthesis, and antioxidant defense systems [9]. Disturbances in metal homeostasis can promote oxidative stress, protein aggregation, and neuronal dysfunction, thereby accelerating neurodegenerative progression [9,33]. Among these metals, iron has received particular attention because excessive iron-mediated redox activity contributes to reactive oxygen species production and cellular injury [34]. In the present study, plasma iron levels were significantly reduced in the Alzheimer’s group and were partially normalized following safranal treatment. Although the precise mechanisms underlying this effect were not investigated, the findings suggest that safranal may contribute to the normalization of altered plasma iron levels in this AD model. This observation is of particular interest given the growing evidence linking altered iron metabolism to cognitive decline, neuronal vulnerability, and the progression of AD pathology [35,36]. Previous studies have shown that saffron bioactives can promote the transcriptional activation of the Nuclear Factor Erythroid 2-Related Factor 2 (Nrf2) pathway and, consequently, increase the expression of cytoprotective enzymes such as Heme Oxygenase-1 (HO-1) [37,38]. By significantly bolstering the cellular antioxidant pool, this Nrf2/HO-1 activation can mitigate systemic oxidative stress and peripheral inflammatory cascades—factors that typically trigger a rise in systemic hepcidin and the subsequent degradation of ferroportin. This mechanism could explain the modulation of plasma iron and cerebral FRAP by safranal observed in our study; however, as these specific parameters were not examined in the current study, further research is required for confirmation.

Oxidative stress represents one of the major downstream consequences of disrupted metal homeostasis and is considered a central mechanism contributing to AD progression [39]. Consistent with this concept, the Alzheimer’s group exhibited a significant reduction in brain total antioxidant capacity as determined by the FRAP assay. Safranal treatment significantly improved FRAP values, indicating enhanced brain total antioxidant capacity. Previous studies have shown that safranal possesses potent free radical-scavenging properties and can protect neuronal cells against oxidative injury and apoptosis [40,41]. Therefore, the improvement in brain total antioxidant capacity observed in the present study may contribute to the behavioral and cellular protective effects associated with safranal administration. The observed alterations in plasma iron levels, alongside decreased cerebral FRAP capacity, reflect peripheral metabolic shifts in this model. While direct brain iron transport was not quantified, the depletion of plasma iron in the untreated AD group may reflect systemic alterations linked to oxidative stress. Our findings suggest that safranal intervention partially corrects plasma iron imbalances and normalizes cerebral FRAP capacity.

Histological and immunohistochemical analyses did not reveal overt structural changes or detectable Aβ accumulation in the hippocampal formations. This critical outcome is consistent with previous landmark reports indicating that the icv-STZ-induced AD model primarily precipitates behavioral impairments, profound oxidative stress, and central metabolic/mitochondrial dysfunction during its early, prodromal stages, typically in the absence of extensive neuronal death or mature extracellular amyloid plaque staging [42,43]. Because our 4-week experimental timeframe explicitly targeted this metabolic window, the severe exploratory and cognitive deficits observed in the untreated Alzheimer’s group likely stem from early synaptic desynchronization and plasma iron imbalances rather than advanced structural necrosis. Consequently, the protective capacity of safranal documented herein is precisely characterized as a functional, metabolic, and cytoprotective stabilization of early-stage prodromal neuropathology, rather than a rescue from macroscopic structural dissolution.

The in vitro findings complement the in vivo results and support the neuroprotective effects of safranal. This calculated IC_50_ value of 5.96 µM for Aβ1-42 in differentiated SH-SY5Y cells is consistent with established neurotoxic profiles in peer-reviewed literature, where micromolar ranges (5–10 µM) are standardly required to induce robust, reproducible apoptotic cascades within a 48 h window [44]. While local soluble oligomer levels in early clinical stages may be lower, this concentration remains physiologically relevant to model the severe, localized cytotoxicity surrounding senile plaques in the advanced clinical microenvironment. Safranal exhibited a dose-specific cytoprotective profile in which therapeutic rescue activity was prominent within the low-dose range (40–60 µM), whereas at higher concentrations, this rescue efficacy progressively declined and cell survival rates dropped, demonstrating that exceeding the optimal therapeutic rescue window limits its beneficial efficacy within this post-treatment paradigm. Hoechst staining revealed typical apoptotic features, including chromatin condensation and nuclear fragmentation, after Aβ exposure, whereas safranal attenuated these alterations, supporting its antiapoptotic potential. Collectively, the concordance between the in vivo and in vitro findings strengthens the evidence supporting the neuroprotective effects of safranal in experimental models of AD.

The present study has several limitations that should be acknowledged. Although we identified a novel and robust association between safranal administration and the restoration of systemic plasma iron levels, specific brain tissue iron concentrations and key central or peripheral molecular iron-regulatory pathways were not examined. While we successfully quantified cerebral total antioxidant capacity via the brain tissue FRAP assay, this specific methodology primarily measures the reducing capacity of aqueous-phase antioxidants, without capturing lipid-soluble components or endogenous enzymatic defenses. Additionally, molecular signaling pathways and transcription factors involved in cell survival and antioxidant defense have not been investigated. Furthermore, our hippocampal histological assessments were primarily qualitative, and individual fluorescent nuclei in the Hoechst assay were not digitally segmented for high-throughput quantification. The absence of histological alterations observed across experimental groups represents a limitation inherent to the early, prodromal phase of the icv-STZ model captured within our 4-week timeframe. Moreover, the lack of a standalone, safranal-only control series in healthy cells represents an in vitro limitation, restricting the definitive differentiation between a failure of the therapeutic rescue window and compound cytotoxicity at elevated doses. Future mechanistic studies tracking central iron homeostasis, advanced enzymatic antioxidant signatures, and molecular transport receptors are warranted to validate these preliminary findings regarding neurodegeneration, employing prolonged experimental timelines.

## 4. Materials and Methods

### 4.1. Ethical Approval

This study was approved by the Gazi University Animal Experiments Local Ethics Committee on 25 September 2024 (Decision No: G.Ü.ET-24.089), and all procedures were performed in accordance with institutional and national guidelines for the care and use of laboratory animals.

### 4.2. Project Support

This study was supported by the Scientific and Technological Research Council of Türkiye (TÜBİTAK) under project code 224S564.

### 4.3. Animals and Experimental Groups

Thirty-five adult male Sprague–Dawley rats (250–300 g) were housed in standard polycarbonate cages under controlled environmental conditions (12 h light/12 h dark cycle, 22 ± 2 °C, 50–60% humidity) with ad libitum access to commercial rodent chow and sterile tap water. Animals were acclimated for 7 days before experimentation.

Rats were randomly assigned to the experimental groups by an independent laboratory personnel who was completely blinded to the study design, hypotheses, and intended treatment groups, thereby ensuring strictly unbiased allocation.

Rats were assigned into five groups (*n* = 7 each):

1. Control: No surgical intervention or pharmacological treatment.
2. Sham: Stereotaxic surgery + icv saline + daily ip saline for 4 weeks.
3. Alzheimer’s: Bilateral icv streptozotocin (3 mg/kg; 2.5 µL per ventricle) + daily ip saline.
4. Safranal: Icv saline + daily ip safranal (0.5 mg/kg/day) for 4 weeks.
5. Alzheimer’s + Safranal: Bilateral icv streptozotocin + safranal (0.5 mg/kg/day) after 24 h recovery.

The single dose of safranal (0.5 mg/kg/day) and the 4-week treatment duration were carefully selected based on previous landmark rodent neurodegeneration literature demonstrating optimal neuroprotective, anti-inflammatory, and antioxidant efficacy in brain tissue without eliciting systemic or behavioral toxicity [45]. Safranal was prepared daily by dissolving the compound in a vehicle configuration consisting of sterile physiological saline (0.9% NaCl) supplemented with 0.5% Tween-80 to maximize solubility and stability. To ensure strict procedural control, both the sham and the untreated Alzheimer’s groups received equivalent volume administrations of this identical saline/Tween-80 vehicle layout throughout the 4-week experimental process.

### 4.4. Stereotaxic Surgery and Icv Injections

Stereotaxic coordinates were determined using the Paxinos and Watson rat brain atlas [46]. Rats were anesthetized with ketamine/xylazine and positioned in a stereotaxic frame. Bilateral injections targeted the lateral ventricles using coordinates defined relative to bregma for the anteroposterior (AP: −0.8 mm) and mediolateral (ML: ±1.5 mm) axes, and relative to the dural surface for the dorsoventral depth (DV: −3.8 mm) [46]. A Hamilton microsyringe (10 μL) delivered the solution slowly; the needle remained in place for 2 min to prevent reflux.

### 4.5. In Vivo Experiments

#### 4.5.1. Behavioral Tests

The open field test assessed locomotor activity, exploratory behavior, and anxiety-like responses [47]. Rats explored a 100 × 100 × 45 cm arena for 5 min. Line crossings, rearing, grooming, defecation, and immobility were recorded. The test was performed twice: baseline and post-treatment.

The novel object recognition test evaluated recognition memory [48]. After habituation, rats explored two identical objects, followed by a test phase with one novel object. Discrimination and recognition indices were calculated:

Discrimination index: (Novel − Familiar)/(Novel + Familiar)

Recognition index: Novel/(Novel + Familiar)

#### 4.5.2. Sacrifice and Sample Collection

After behavioral testing, rats were sacrificed under deep ketamine/xylazine anesthesia. Blood samples were collected immediately via cardiac puncture to obtain plasma. Concurrently, the cranium was quickly opened, and the whole brain was excised on an ice-cold dissection plate. One hemisphere was immediately fixed in 10% neutral buffered formalin for downstream histological and immunohistochemical hippocampal staging (CA1, CA3, and DG regions). The contralateral hemisphere was collected as whole-brain tissue and stored at −80 °C until total antioxidant capacity quantification via the FRAP assay.

#### 4.5.3. Histological Analysis

The fixed cerebral hemispheres were dehydrated, embedded in paraffin, and sectioned coronally at a thickness of 5 μm. Hematoxylin–Eosin (H&E) staining was utilized to evaluate general hippocampal cytoarchitecture within the cornu ammonis 1 (CA1), CA3, and dentate gyrus (DG) regions. Hippocampal tissue organization was evaluated qualitatively by a blinded investigator.

Amyloid-related pathology and immunoreactivity profiles were assessed immunohistochemically using the MOAB-2 primary antibody (sc-374527, Santa Cruz, CA, USA). Sections underwent standardized heat-induced antigen retrieval, endogenous peroxidase blocking, and serum blocking, followed by primary antibody incubation, biotinylated secondary antibody tracking, streptavidin–HRP conjugation, and 3,3′-diaminobenzidine (DAB) chromogen visualization. MOAB-2 staining density and distribution patterns within the CA1, CA3, and DG subregions were evaluated via qualitative visual microscopy assessment conducted by a blinded investigator.

### 4.6. ICP-MS Metal Analysis

Plasma concentrations of iron, copper, and zinc were quantified using Inductively Coupled Plasma–Mass Spectrometry (ICP-MS) (NexION^®^ 2000, PerkinElmer, Waltham, MA, USA) to evaluate metal levels associated with AD and the potential modulatory effects of safranal. Blood samples were collected into metal-free tubes immediately after sacrifice and centrifuged at 4500 rpm for 10 min to obtain plasma. All samples were stored at −80 °C until analysis to prevent metal degradation or contamination.

Prior to analysis, plasma samples were thawed on ice and subjected to acid digestion. A 100 μL aliquot of plasma was mixed with 900 μL of ultrapure 65% nitric acid (HNO_3_) in metal-free polypropylene tubes. Samples were incubated at 60 °C for 2 h to ensure complete digestion. After cooling to room temperature, digested samples were diluted with ultrapure deionized water (18.2 MΩ·cm) to a final volume of 10 mL, resulting in a precise 100-fold dilution factor for analysis. All reagents and consumables were certified for trace metal analysis to minimize background contamination.

Metal quantification was performed using a high-resolution ICP-MS system (NexION^®^ 2000, PerkinElmer, Waltham, MA, USA) equipped with a collision/reaction cell using helium gas to reduce polyatomic interferences. The instrument was calibrated using multi-element standard solutions containing iron, copper, and zinc. Calibration curves were generated using a 6-point standard concentration range spanning 100–10,000 µg/L for Fe, 10–1000 µg/L for Cu, and 10–1000 µg/L for Zn. These linear calibration ranges strictly covered the matrix-matched concentrations of all analyzed samples, ensuring that no sample concentration fell outside the validated quantification limits.

To correct for matrix effects and signal drift, Rhodium (^103^Rh) and Yttrium (^89^Y) were utilized as internal standards and added to all blanks, standards, and samples at a final concentration of 10 μg/L. Instrument parameters (plasma power, nebulizer gas flow, dwell time) were optimized daily to achieve maximum sensitivity and stability.

Procedural blanks were processed alongside samples to monitor background contamination. Analytical accuracy and precision were verified by analyzing a certified reference material (NIST SRM 1598a—Animal Serum). The recovered concentrations for all target metals were within 92–104% of the certified values. The limit of detection (LOD) and limit of quantification (LOQ) were calculated based on 3 and 10 times the standard deviation of the procedural blanks, respectively (Table 6). Duplicate measurements were performed for each sample, and the relative standard deviation (RSD) was consistently below 5%. Final plasma metal concentrations were calculated back to the original undiluted sample using the 100-fold dilution factor and expressed in mg/L for iron, μg/dL for copper, and μg/mL for zinc to adhere to standard reporting formats (Table 6).

### 4.7. FRAP Assay

Total antioxidant capacity was measured using the FRAP assay (EBCK225M, Elabscience Biotechnology Inc., Houston, TX, USA). Brain tissue homogenates were incubated with FRAP working solution at 37 °C for 10 min, and absorbance was read at 593 nm. Concentrations were calculated using standard curves.

### 4.8. In Vitro Experiments

#### 4.8.1. Cell Culture and Treatment Protocols

Human SH-SY5Y neuroblastoma cells (ATCC^®^ CRL-2266™) were cultured in DMEM/F12 medium supplemented with 10% fetal bovine serum (FBS) and 1% penicillin–streptomycin in a humidified atmosphere containing 5% CO_2_ at 37 °C. To induce advanced neuronal differentiation and promote the extension of complex neuritic networks, cells were incubated with 10 μM all-trans retinoic acid (RA) for a total duration of 10 days, with the differentiation medium refreshed every 48 h [49,50].

To establish the in vitro AD model, differentiated neuroblastoma cells were initially exposed to graded concentrations of Aβ1-42 (0.83, 1.65, 3.13, 6.25, 12.5, 25, and 50 µM) dissolved in 1% DMSO for 48 h [44]. Cell viability outcomes were subsequently quantified via the MTT assay to model the concentration-dependent neurotoxic screening and calculate the half-maximal inhibitory concentration (IC_50_) value at the 48 h endpoint.

To evaluate the post-toxicity therapeutic rescue capacity of safranal rather than a mere prophylactic or preventative action, an in vitro sequential post-treatment paradigm was executed. Differentiated cells were initially pre-incubated with the designated neurotoxic Aβ1-42 baseline (~5.96 µM) for 48 h to fully establish robust cellular neurotoxicity. Following this initial insult phase, the amyloid-containing media was completely evacuated, cells were carefully washed to remove free peptides, and a full range of graded therapeutic doses of safranal (40, 60, 80, 100, 120, 140, and 160 µM) were subsequently administered in fresh media for an additional 48 h to delineate post- toxicity survival windows. The concentration range of safranal was carefully selected based on literature establishing the compound’s therapeutic windows in SH-SY5Y cells [30].

#### 4.8.2. MTT Assay

MTT assays were performed using three independent biological replicates, with each experimental condition plated in triplicate. A vehicle control containing ≤0.1% DMSO was included to rule out solvent toxicity. Following the treatment period, cells were incubated with MTT solution (0.5 mg/mL) for 3 h at 37 °C, after which the resulting formazan crystals were solubilized in DMSO, and absorbance was measured at 570 nm [51].

#### 4.8.3. Hoechst Apoptosis Assay

To provide a qualitative morphological assessment of nuclear architecture and apoptosis, cells were fixed with 4% paraformaldehyde for 15 min at room temperature and stained with Hoechst 33258 dye (HY-15558, MedChemExpress, Monmouth Junction, NJ, USA) in accordance with standard protocols [52]. Qualitative visual imaging of representative fields was performed using a fluorescence microscope (Carl Zeiss Microscopy GmbH, Oberkochen, Germany) to evaluate changes in nuclear condensation and apoptotic fragmentation across experimental conditions.

### 4.9. Statistical Analysis

Data were analyzed using GraphPad Prism 8 (GraphPad Software, San Diego, CA, USA). Normality was assessed using the Shapiro–Wilk test, and homogeneity of variance was verified via Levene’s test across all datasets prior to running parametric evaluations. For behavioral parameters involving longitudinal assessments at baseline and final time points (Open Field Test), as well as the Discrimination Index across different trial phases (Novel Object Recognition), data were analyzed using Two-Way Repeated Measures ANOVA (Group × Time/Phase) to account for within-subject correlations, followed by Sidak’s multiplicity-adjusted post hoc test. For single-endpoint parameters containing no longitudinal tracking (Recognition Index, plasma metal levels, and FRAP values), statistical evaluations were conducted via One-Way ANOVA followed by Tukey’s multiplicity-adjusted post hoc test. Results with *p* < 0.05 were considered statistically significant. Data are presented as mean ± standard deviation.

## 5. Conclusions

Safranal demonstrated neuroprotective effects in both in vivo and in vitro AD models by improving behavioral performance, partially correcting plasma iron levels, enhancing cerebral FRAP capacity, and mediating cytoprotective rescue windows against Aβ-induced cytotoxicity and apoptosis. Although no overt histopathological or amyloid-related alterations were detected in the early-stage STZ model, safranal improved plasma iron levels and cerebral total antioxidant status. The convergence of behavioral, biochemical, and cellular findings supports the neuroprotective potential of safranal in experimental models of AD. Nevertheless, further studies are required to clarify the underlying molecular mechanisms and validate these findings in more advanced disease models. Collectively, the present results suggest that safranal may represent a promising natural compound for mitigating AD-related neurodegenerative processes.

## Figures and Tables

**Table 1:** Line crossing counts in the periphery and center of rats in the OFT at baseline and final time points.

Experimental Groups (*n* = 7)	Line Crossing—Periphery		Line Crossing—Center	
	Baseline	Final	Baseline	Final
**Control**	73.00 ± 6.65	73.00 ± 14.05	16.85 ± 5.16	15.00 ± 4.37
**Sham**	73.42 ± 8.87	76.28 ± 6.88	21.28 ± 4.62	17.14 ± 3.64
**Alzheimer’s**	71.57 ± 5.23	62 ± 5.93 *^,#,&^	13.42 ± 4.89	7.43 ± 2.06 *^,#,&,$^
**Safranal**	75.28 ± 6.22	77.57 ± 6.02	21.28 ± 5.20	24.71 ± 4.39 *^,#^
**Alzheimer’s + Safranal**	74.86 ± 3.78	67.57 ± 7.18	19.42 ± 5.39	14.57 ± 3.84

Line Crossing—Periphery: Interaction F(4, 30) = 3.093, *p* = 0.0303; Main effect of Time F(1, 30) = 2.599, *p* = 0.1174; Main effect of Group F(4, 30) = 1.896, *p* = 0.1370; Residual MSE = 36.96. Line Crossing—Center: Interaction F(4, 30) = 3.175, *p* = 0.0274; Main effect of Time F(1, 30) = 8.194, *p* = 0.0076; Main effect of Group F(4, 30) = 9.609, *p* < 0.0001; Residual MSE = 15.40. Data are expressed as Mean ± SD (*n* = 7 per group). Statistical evaluations were conducted via Two-Way Repeated Measures ANOVA followed by Sidak’s multiplicity-adjusted post hoc test, confirming no statistically significant baseline differences between any experimental groups (*p* > 0.05) prior to surgical interventions. * *p* < 0.05 vs. Control group; ^#^ *p* < 0.05 vs. Sham group; ^&^ *p* < 0.05 vs. Safranal group; ^$^ *p* < 0.05 vs. Alzheimer’s + Safranal group using Sidak’s multiplicity-adjusted post hoc test.

**Table 2:** Rearing frequency and rearing duration (seconds) of rats in the OFT at baseline and final time points.

Experimental Groups (*n* = 7)	Rearing—Frequency		Rearing—Duration	
	Baseline	Final	Baseline	Final
**Control**	11.43 ± 1.92	14.28 ± 2.81	24.86 ± 4.22	29.71 ± 5.84
**Sham**	15.57 ± 4.43	11.71 ± 3.14	29.57 ± 8.55	23.71 ± 4.46
**Alzheimer’s**	12.14 ± 2.23	7.85 ± 1.12 *^,&^	24.14 ± 5.51	15.28 ± 2.31 *^,#,&^
**Safranal**	11.43 ± 2.55	12.00 ± 2.32	24.86 ± 4.94	23.00 ± 4.03
**Alzheimer’s + Safranal**	11.86 ± 2.23	8.14 ± 2.29 *	24.00 ± 6.16	17.71 ± 3.76 *

Rearing—Frequency: Interaction F(4, 30) = 4.443, *p* = 0.0061; Main effect of Time F(1, 30) = 6.111, *p* = 0.0193; Main effect of Group F(4, 30) = 4.695, *p* = 0.0046; Residual MSE = 8.138. Rearing—Duration: Interaction F(4, 30) = 3.434, *p* = 0.0200; Main effect of Time F(1, 30) = 7.772, *p* = 0.0091; Main effect of Group F(4, 30) = 4.595, *p* = 0.0052; Residual MSE = 29.18. Data are expressed as Mean ± SD (*n* = 7 per group). Statistical evaluations were conducted via Two-Way Repeated Measures ANOVA followed by Sidak’s multiplicity-adjusted post hoc test, confirming no statistically significant baseline differences between any experimental groups (*p* > 0.05) prior to surgical interventions. * *p* < 0.05 vs. Control group; ^#^ *p* < 0.05 vs. Sham group; ^&^ *p* < 0.05 vs. Safranal group using Sidak’s multiplicity-adjusted post hoc test.

**Table 3:** Defecation count, immobility frequency, and immobility duration (seconds) of rats in the OFT at baseline and final time points.

Experimental Groups (*n* = 7)	Defecation		Immobility—Frequency		Immobility—Duration	
	Baseline	Final	Baseline	Final	Baseline	Final
**Control**	3.28 ± 1.27	1.86 ± 1.80	0.43 ± 0.29	0.86 ± 0.79	0.93 ± 0.19	3.28 ± 3.01
**Sham**	2.14 ± 1.80	1.00 ± 0.93	1.90 ± 1.47	0.57 ± 0.32	4.43 ± 2.65	2.43 ± 2.03
**Alzheimer’s**	1.00 ± 0.75	0.57 ± 0.72	0.72 ± 0.49	1.14 ± 0.99	3.57 ± 2.03	5.71 ± 4.23
**Safranal**	1.71 ± 1.38	2.00 ± 1.85	0.58 ± 0.33	1.00 ± 0.92	2.71 ± 1.57	4.44 ± 3.32
**Alzheimer’s + Safranal**	3.14 ± 1.80	0.86 ± 0.83	0.43 ± 0.29	0.71 ± 0.20	2.79 ± 1.56	5.71 ± 4.47

Defecation: Interaction F(4, 30) = 1.691, *p* = 0.1782; Main effect of Time F(1, 30) = 8.813, *p* = 0.0058; Main effect of Group F(4, 30) = 2.350, *p* = 0.0768; Residual MSE = 1.986. Immobility—Frequency: Interaction F(4, 30) = 0.7234, *p* = 0.5828; Main effect of Time F(1, 30) = 1.362, *p* = 0.2524; Main effect of Group F(4, 30) = 0.3220, *p* = 0.8610; Residual MSE = 0.6714. Immobility—Duration: Interaction F(4, 30) = 1.189, *p* = 0.3356; Main effect of Time F(1, 30) = 3.372, *p* = 0.0762; Main effect of Group F(4, 30) = 0.6558, *p* = 0.6274; Residual MSE = 12.35. Data are expressed as mean ± SD (*n* = 7 per group). Statistical evaluations were performed using Two-Way Repeated Measures ANOVA followed by a post hoc test with Sidak’s correction for multiple comparisons; it was confirmed that there were no statistically significant differences in between-group comparisons within the same time points (*p* > 0.05).

**Table 4:** Grooming frequency and grooming duration (seconds) of rats in the OFT at baseline and final time points.

Experimental Groups (*n* = 7)	Grooming—Frequency		Grooming—Duration	
	Baseline	Final	Baseline	Final
**Control**	8.71 ± 0.88	6.71 ± 1.03	26.14 ± 3.76	23.14 ± 4.42
**Sham**	7.71 ± 1.57	7.14 ± 1.80	24.42 ± 5.95	20.57 ± 6.71
**Alzheimer’s**	7.43 ± 1.59	8.14 ± 1.64	25.28 ± 3.95	24.00 ± 2.39
**Safranal**	8.28 ± 2.25	7.86 ± 2.23	24.28 ± 4.43	22.28 ± 4.94
**Alzheimer’s + Safranal**	8.86 ± 1.81	8.43 ± 2.44	26.14 ± 5.30	20.57 ± 4.59

Grooming—Frequency: Interaction F(4, 30) = 1.267, *p* = 0.3047; Main effect of Time F(1, 30) = 2.002, *p* = 0.1674; Main effect of Group F(4, 30) = 0.6038, *p* = 0.6629; Residual MSE = 2.576. Grooming—Duration: Interaction F(4, 30) = 0.6300, *p* = 0.6449; Main effect of Time F(1, 30) = 11.13, *p* = 0.0023; Main effect of Group F(4, 30) = 0.3241, *p* = 0.8596; Residual MSE = 15.53. Data are expressed as Mean ± SD (*n* = 7 per group). Statistical evaluations were conducted via Two-Way Repeated Measures ANOVA followed by Sidak’s multiplicity-adjusted post hoc test, confirming no statistically significant differences between any of the experimental groups (*p* > 0.05).

**Table 5:** Recognition memory performance of rats in the Novel Object Recognition (NOR) test across experimental phases.

Experimental Groups (*n* = 7)	Identical Objects Phase	Different Objects Phase	
	Discrimination Index (%)	Discrimination Index (%)	Recognition Index (%)
**Control**	13.33 ± 3.2	36.66 ± 11.2	69.43 ± 14.9
**Sham**	16.66 ± 1.9	40.00 ± 9.1	70.71 ± 10.5
**Alzheimer’s**	20.00 ± 2.2	12.66 ± 7.5 *^,#,&,$^	55.29 ± 11.3 *^,#,&^
**Safranal**	24.57 ± 3.9	39.33 ± 9.6	69.14 ± 9.8
**Alzheimer’s + Safranal**	14.66 ± 1.7	23.33 ± 9.9 *^,#,&^	60.56 ± 7.2 *^,#,&^

Discrimination Index: Interaction F(4, 30) = 8.132, *p* = 0.0001; Main effect of Phase F(1, 30) = 46.10, *p* < 0.0001; Main effect of Group F(4, 30) = 11.43, *p* < 0.0001; Residual MSE = 79.35. Recognition Index: One-Way ANOVA F(4, 30) = 6.020, *p* = 0.001; Residual MSE = 76.04. Data are expressed as Mean ± SD (*n* = 7 per group). Statistical evaluations were conducted via Two-Way Repeated Measures ANOVA followed by Sidak’s multiplicity-adjusted post hoc test, confirming no statistically significant differences during the identical objects phase (*p* > 0.05), while highly significant alterations emerged during the different objects phase. The Recognition Index (RI) during the different objects phase was analyzed using One-Way ANOVA followed by Tukey’s post hoc test. * *p* < 0.05 vs. Control group; ^#^ *p* < 0.05 vs. Sham group; ^&^ *p* < 0.05 vs. Safranal group; ^$^ *p* < 0.05 vs. Alzheimer’s + Safranal group.

**Table 6:** Analytical Performance and Validation Parameters of the ICP-MS Method.

Metals	Calibration Range (Instrumental, µg/L)	Linear Regression (R^2^)	LOD (µg/L)	LOQ (µg/L)	Internal Standard	CRM Recovery (%)	Final Reported Units (in Plasma)
**Iron (Fe)**	100–10,000	>0.9995	0.45	1.50	^89^Y	98.5%	mg/L
**Copper (Cu)**	10–1000	>0.9998	0.12	0.40	^103^Rh	101.2%	µg/dL
**Zinc (Zn)**	10–1000	>0.9996	0.28	0.95	^103^Rh	97.4%	µg/mL

Fe: Iron; Cu: Copper; Zn: Zinc; R^2^: Coefficient of determination; LOD: Limit of Detection; LOQ: Limit of Quantification; CRM: Certified Reference Material. Instrumental calibration ranges and LOD/LOQ values represent the direct solution concentrations measured by the NexION^®^ 2000 system before applying the 100-fold plasma dilution factor to calculate final plasma concentrations.

## Data Availability

The datasets generated and analyzed during the current study are available from the corresponding author upon reasonable request.

## Abbreviations

The following abbreviations are used in this manuscript:

AD	Alzheimer’s Disease
Aβ	Amyloid-β
CA	Cornu Ammonis
CRM	Certified Reference Material
Cu	Copper
DAB	3,3′-Diaminobenzidine
DF	Degrees of Freedom
DG	Dentate Gyrus
DI	Discrimination Index
DMEM/F12	Dulbecco’s Modified Eagle’s Medium and Ham’s F-12 Nutrient Mixture
DMSO	Dimethyl Sulfoxide
F F-ratio	Fisher’s Analysis of Variance Statistic
FBS	Fetal Bovine Serum
Fe	Iron
FRAP	Ferric Reducing Antioxidant Power
H&E	Hematoxylin–Eosin
HO-1	Heme Oxygenase-1
HRP	Horseradish Peroxidase
ICP-MS	Inductively Coupled Plasma Mass Spectrometry
IC_50_	Half-maximal Inhibitory Concentration
Icv	Intracerebroventricular
icv-STZ	Intracerebroventricular Streptozotocin
ip	Intraperitoneal
LOD	Limit of Detection
LOQ	Limit of Quantification
MSE	Mean Squared Error
MTT	3-(4,5-Dimethylthiazol-2-yl)-2,5-diphenyltetrazolium Bromide
NaCl	Sodium Chloride
NOR	Novel Object Recognition
Nrf2	Nuclear Factor Erythroid 2-related Factor 2
OFT	Open Field Test
PBS	Phosphate-buffered Saline
RA	Retinoic Acid
RI	Recognition Index
RM ANOVA	Repeated Measures Analysis of Variance
ROS	Reactive Oxygen Species
SD	Standard Deviation
STZ	Streptozotocin
Zn	Zinc
